# Diazotrophy in Alluvial Meadows of Subarctic River Systems

**DOI:** 10.1371/journal.pone.0077342

**Published:** 2013-11-06

**Authors:** Thomas H. DeLuca, Olle Zackrisson, Ingela Bergman, Beatriz Díez, Birgitta Bergman

**Affiliations:** 1 School of the Environmental and Forest Sciences, University of Washington, Seattle, Washington, United States of America; 2 Institute for Subarctic Landscape Research, Torgatan 20, Arjeplog, Sweden; 3 Department of Molecular Genetics and Microbiology, Faculty of Biological Sciences, Pontificia Universidad Católica de Chile, Alameda 340, C.P. 651 3677, Santiago, Chile; 4 Department of Ecology, Environment and Plant Sciences, Stockholm University, Stockholm, Sweden; University of New South Wales, Australia

## Abstract

There is currently limited understanding of the contribution of biological N_2_ fixation (diazotrophy) to the N budget of large river systems. This natural source of N in boreal river systems may partially explain the sustained productivity of river floodplains in Northern Europe where winter fodder was harvested for centuries without fertilizer amendments. In much of the world, anthropogenic pollution and river regulation have nearly eliminated opportunities to study natural processes that shaped early nutrient dynamics of large river systems; however, pristine conditions in northern Fennoscandia allow for the retrospective evaluation of key biochemical processes of historical significance. We investigated biological N_2_ fixation (diazotrophy) as a potential source of nitrogen fertility at 71 independent floodplain sites along 10 rivers and conducted seasonal and intensive analyses at a subset of these sites. Biological N_2_ fixation occurred in all floodplains, averaged 24.5 kg N ha^−1^ yr^−1^ and was down regulated from over 60 kg N ha^−1^ yr^−1^ to 0 kg N ha^−1^ yr^−1^ by river N pollution. A diversity of N_2_-fixing cyanobacteria was found to colonize surface detritus in the floodplains. The data provide evidence for N_2_ fixation to be a fundamental source of new N that may have sustained fertility at alluvial sites along subarctic rivers. Such data may have implications for the interpretation of ancient agricultural development and the design of contemporary low-input agroecosystems.

## Introduction

Alluvial floodplains (meadows and wetlands) of large river systems are among the most productive ecosystems on Earth [1]–[4], yet floodplains are dynamic landscapes which may function as both a sink or source of nutrients [5], [6]. Floodplain fertility is often broadly ascribed to nutrient deposition during flooding events, and the natural productivity of floodplains yields immense production of grasses, sedges and herbs. This makes these floodplain landscapes a foci for grazing ungulates [7] and the cradle of agrarian culture [8]–[10]. Similar to their ancient farming predecessors in Euphrates, Tigris and the Nile, farmers settling the sub-arctic regions in the interior of northern Europe selectively exploited floodplains and also constructed intricate water fed meadow systems to increase fodder production [11]. These productive meadows are a dramatic contrast to the surrounding often species poor, slow growing and low productivity coniferous forests. Alluvial floodplains at northern latitudes (Arctic, subarctic and boreal) with their long winter seasons were particularly valuable resources used for cattle farming and for the harvest of winter fodder until the middle of the 20^th^ Century [11], [12]. The unfertilized, seasonally flooded meadows exhibited exceptional productivity resilience in spite of annual harvests over extended periods of time [12], [13]. Flooded meadows were the backbone of fodder production until commercial fertilisers were introduced and production switched to ploughed fields on higher ground where flooding did not interfere with sewing or harvesting of crops [11], [14], [15]. The scientific basis for this high and sustained productivity of alluvial meadows has been intensively debated in the past [7], but any meaningful explanation has remained elusive [4], [16]. Today, pollution, agricultural and urban land development, and river regulation along most large river systems in Europe [17]–[19] have made it nearly impossible to retrospectively investigate mechanisms that dictated the intrinsic productivity of these agricultural settings.

It is well established that water, *per se*, seldom limits growth of vascular plants at northern boreal sites, instead N availability is considered to be a key factor limiting growth [20], with little or no consideration being given to vital processes such as biological N_2_ fixation in this context [21]. Snow melt water that floods these alluvial meadows contains very little inorganic N and available N concentrations in flood waters in subarctic landscapes are extremely low [22] and cannot account for the sustained annual N demand for plant growth on alluvial meadows [23]. Biological N_2_ fixation (diazotrophy) is the primary source of ‘new’ N in many natural globally spread oligotrophic ecosystems [24] and the profound role of N_2_-fixing prokaryotes in both terrestrial and marine biogeochemicals cycles of N is by now well-established [5], [17], [25]. In spite of the plant species richness associated with boreal alluvial meadows [26], these environments lack any known quantity of N_2_-fixing bacterial-plant associations [27] that could help explain the origin of the N required for plant growth and seasonal harvest.

To date there has been no significant effort to evaluate the diazatrophy in floodplains as a potential N contribution to riverine ecosystems. We therefore hypothesized that N_2_ fixation may represent a source of ‘new’ N following spring floods and that free-living prokaryotic N_2_-fixers (diazotrophs), with focus on the photoautotrophic cosmopolitan cyanobacteria, may inhabit the light exposed soil surfaces and plant debris. Herein, we provide the first large-scale effort to quantify biological N_2_ fixation in floodplains of large river systems in northern Europe. To determine the possible role of diazotrophic N_2_ fixation in supplying ecosystem N in subarctic floodplains, replicate surface organic samples from 71 seasonally flooded meadow sites along 10 unregulated free flowing subartic rivers between latitudes 64–68° N in northern Sweden were collected and analyzed for nitrogenase (the enzyme complex catalyzing N_2_ fixation) activity and potential diazotrophic actors were identified. These studies were conducted at latitudes where there is still low anthropogenic N deposition [28] and limited agricultural or urban N pollution, factors otherwise known to severely down-regulate biological N_2_ fixation [29], [30].

## Methods and Materials

Studies were conducted at meadow sites along 10 large, unregulated river systems in northern Sweden (see Supporting Information). Vegetation on these floodplains was dominated by mosses, grasses, sedges (*Carex* spp.), herbs and willow (*Salix* spp.) trees. The studies were conducted along 1^st^, 2^nd^, and 3^rd^ order (1^st^ order = rivers draining into the Baltic Sea, 2^nd^ and 3^rd^ order are primary and secondary tributaries) rivers in northern Sweden between latitudes 64–68° N and longitudes 16–24° E (Fig. S1). All study sites are on open, alluvial meadows (Fig. S2) that are dominated by various mosses (*Drepanocladus spp* and *Scorpidium* spp.) with a common presence of grasses (e.g. *Deschampsia* spp.), sedges (*Carex* spp.), and various herbs (e.g. *Sparganium* spp., *Eriophorum* spp., *Ranunculus* spp.). Margins of meadows were often dominated by willows (*Salix* spp.) and occasionally fine birchwood (*Betula pubescence* L.).

Four primary studies are described below: (1) A broad analysis of diazatrophy conducted across 71 independent river floodplains throughout northern Fennoscandia; (2) A river gradient study conducted on 17 meadows sampled on three occasions along one river (Torne River); (3) An intensive seasonal study conducted for three consecutive years along four river segments of the Lais, Pite and Vindel (two sites) Rivers; (4) A small scale study was conducted on N fertilization and clipping influence on N fixation at one location along Vindel River. Together, these studies allow us to effectively quantify biological N_2_ fixation on large river floodplains in northern Sweden. All sites are natural reserves and protected areas along rivers in Sweden and were accessed according to the open land policies of the government of Sweden (*allemansrätten).* The collection of soil and plant tissue samples on public lands and reserves was allowed under an existing permit under the authority of Länsstyrelsen i Norrbottens län and held by Professor Marie-Charlotte Nilsson (SLU-Umeå) and extended to research associates including members of the Institute for Subarctic Landscape Research in Arjeplog Sweden.

### Assessment of Diazotrophy in Alluvial Meadows of Rivers in Northern Fennoscandia

Twelve replicate samples were collected at 71 individual alluvial meadow sites along 10 rivers (Fig. S1) in northern Sweden between latitudes 64–68 N and longitudes 16–24 E. Atmospheric deposition of N is very low in this region (1–2 kg N ha^−1^ yr^−1^) and farmland N runoff is minimal to non-existent [22], [28]. To assess N_2_ fixation activity in alluvial meadows, replicate samples composed of sediment and vegetative detritus were collected by removing a 2.5 cm diameter core to a depth of 2 cm deep and placing them directly into 60 ml polyethylene centrifuge tubes. Nitrogen fixation rates by assaying nitrogenase activity by use of the acetylene reduction method [31]. Briefly, sediment cores in 60 ml culture tubes were sealed with a rubber septum (Subaseal) and 10% of air was removed from the headspace using a syringe and replaced with reagent grade acetylene. The tubes were allowed to incubate under natural field conditions over a 24-h period. A 1-ml sample of the headspace was then removed using a gas-tight syringe and analysed for ethylene concentration using a gas chromatograph equipped with a flame ionization detector and a Poropak T column. Acetylene reduction rates were tested for linearity over a 24 hour period with ethylene production measured at 0, 0.25, 0.5, 1, 3, 6, 9, 12, and 24 hours and plotted as nmol ethylene tube^−1^ versus time. Ethylene production over the 24 hour period was found to be linear (R^2^ = 0.992, Y = 116.6+5.61).

### Quantification of N_2_ Fixation Rate and Seasonal N Fixation Pattern

An assessment of season-long N_2_ fixation rates was conducted at a total of four sites on three rivers (Lais, Vindel, Pite Rivers) in northern Sweden. Surface soil samples were collected as described above at snow melt and every two weeks throughout the summer and autumn until snow accumulation in November. Twelve replicate samples were randomly collected from alluvial sediments at each of the four sites on a total of 15, 13, and 10 sampling periods in 2008, 2009, 2010 respectively (over 1700 samples total). Samples were collected and analysed for nitrogenase activity (acetylene reduction) as described above. Tubes were field incubated over a 24-h period. Each value was converted to N_2_ fixed based on a ratio of 2.5 moles acetylene reduced for each mole of N_2_ reduced (see below), weighted by time since last measurement and total N fixed calculated as the sum of N fixed during each time period.

Acetylene reduction rates for surface organic samples (sediment, plant detritus, and microbial biofilms) were calibrated to estimate N_2_ fixation rates by using ^15^N_2_ gas [32]. Parallel sediment and moss samples were placed into 20 mL tubes. Ten percent of the headspace was removed using a gas-tight syringe and replaced with acetylene. Samples were then allowed to incubate for 24 h at 25°C and approximately half of full sunlight. Ethylene concentration in the headspace was measured. Tubes were opened and the acetylene was allowed to escape. The tubes were then capped and again 10% of the headspace removed and replaced with ^15^N_2_ gas enriched at 98% ^15^N_2_. Control tubes containing no moss were tested for actual ^15^N_2_ content in the tubes. Moss samples were dried at 60°C, ground in a ball mill, and analyzed for ^15^N enrichment on a continuous flow combustion analyzer and isotopes ratio mass spectrometer (Duke University Stable Isotopes Laboratory, Durham, NC, USA). Sediment and moss samples collected from the alluvial meadows were found to reduce acetylene at a ratio of 2.5 moles of ethylene per mole N_2_.

### River Elevation Gradient Study

Alluvial meadow sediment samples were collected as described above at 15 locations distributed north to south along a single river system (Torne) in northern Sweden. The sampling covered a total river length of 386 km and an elevation gradient from 368 m to 0 m. This allowed us to evaluate the influence of river elevation and land use on N fixation rates in alluvial meadows. As river elevation declines, the extent and degree of agricultural land use and urban development increases. Samples were collected and analyzed for acetylene reduction on four separate occasions in 2009 and 2010. In the summer of 2010 we evaluated *in-situ* N mineralization at 15 locations along the Torne River, at sites associated with regular N_2_ fixation measurements over the previous two years.

### Resin Sorbed Inorganic N Along the Torne River

*In-situ* N mineralization was accomplished by inserting mesh capsules containing 1 g of mixed bed anion/cation ionic resin (UNIBEST, Walla Walla, WA) into surface sediments in June 2010. Resin capsules were then collected two months later in August, and inorganic N (NH_4_^+^ and NO_3_^−^) were extracted from ionic resin capsules by placing the resin capsules into 10 ml of 2 M KCl in a 50 ml centrifuge tube, shaking for 30 minutes, and then decanting the extract into a clean storage bottle. This process was repeated two additional times to create a total extractant volume of 30 ml. Total NH_4_^+^ and NO_3_^−^ were analyzed on these extracts by using a 96 microtiter plate reader using the Berthelot reaction for NH_4_^+^ analysis and the salycilate method for NO_3_^−^ analysis.

### N_2_ Fixation Along a Latitudinal Gradient in Southern Fennoscandia

Alluvial meadow sediment samples were collected and analyzed for nitrogenase activity (acetylene reduction) as described above at seven locations in southern Norway and Sweden (62° N to 65.3°N). This provided a latitudinal gradient for N2 fixation at southern latitudes where N deposition rates and river N enrichment from human effluent and agricultural practices are greater [28].

### Identification of Cyanobacteria

For light microscopy identification, subsamples of surface organic layers collected at sample sites on the Lais, Pite and Vindel Rivers were fixed and stored in 2.5% glutaraldehyde until use. Representative photomicrographs were acquired after analyzing the samples using light microscopy (Olympus BH-2 or Zeiss Axiovert), equipped with a digital camera. For the genetic identification of cyanobacteria (based on 16S rRNA gene) and diazotrophic cyanobacteria (based on *nifH* gene), nucleic acids were extracted from the samples as described previously [33]. Nucleic acid extracts were stored at –80°C until analyzed. The quality and quantity of the DNA was determined spectrophotometrically using a Nanodrop instrument (Saveen & Werner AB, Sweden) and verified by agarose gel electrophoresis. PCR amplifications were performed using Taq DNA polymerase (Invitrogen) and the cyanobacterial-specific 16S rDNA oligonucleotide primers CYA106F (with a 40 nucleotide GC clamp at the 5′ end) and CYA781R [34], and the cyanobacterial-specific *nif*H oligonucleotide primers CNF (with a 40 bp GC clamp at the 5′ end [34]; and CNR [35], which amplify about 675 bp and 359 bp, respectively. PCR and DGGE conditions followed previous protocols [34], [36]. The dominant DGGE bands were excised from the gels (including several bands occupying the same position in different samples), PCR re-amplified and sequenced (Macrogen, Korea). The DGGE generated sequences were aligned in Bioedit using ClustalW (Tom Hall, Ibis Therapeutics, Carlsbad, USA) and were manually corrected. All sequences were subjected to BLAST searches [37] (www.ncbi.nlm.nih.gov/blast), and the closest relatives obtained from GenBank were included in the subsequent phylogenetic analysis and reconstructions (Fig. S3). Only sequences from published studies or culture collections were included. The cyanobacterial 16S rRNA- and *nif*H-DGGE sequences and the corresponding sequences for reference taxa were used for phylogenetic reconstructions. MrAIC Test (http://www.abc.se/~nylander/mraic/mraic.html) was used to search for the best nucleotide substitution model (GTRIG and TrNIG, respectively). Likelihood scores under different models were estimated using PHYML [38]. Branch support was measured by bootstrap analysis with 1000 bootstrap replicates. The sequences generated in this study are deposited in GenBank under the accession numbers: DGGE-16S rRNA (JN159939–JN159948); DGGE-*nif*H (JN159949–JN159969).

### Biomass Analyses, N Fertilization and Clipping Influence on N_2_ Fixation

A field experiment was performed at one river segment near the area denoted Björkheden along the Vindel River in northern Sweden. Field plots measuring 1 m^2^ were established in a uniform stand of sedge (*Carex* spp.) and treated with: (1) no treatment, control; (2) 4.25 kg N ha^−1^ as NH_4_NO_3_; 25.5 kg N ha^−1^ as NH_4_NO_3_; or vegetation clipped and removed to simulate harvesting of winter fodder. Each treatment was replicated 12 times and randomly assigned to the study area. Sediment samples were collected from the plot area on two separate occasions 30 and 45 days after treatment. Nitrogen capital in river sediments at Björkheden was assessed by collecting replicate sediment cores (10 cm diameter by 15 cm depth) at three sites. The contents of each core were dried, weighed and bulk density calculated by dividing mass by volume. Sediment samples were ground to pass a 76 µm sieve and analysed for total N by dry combustion analyser (LECO CN analyser, St Joeseph, Michigan). Total N mass per unit area was calculated by multiplying depth by density by N concentration. Samples were analyzed for acetylene reduction activity as described above. Data were analyzed using univariate analysis of variance (SPSS).

Vegetative biomass plots were established at 10 individual study sites along the Vindel, Pite, and Lais Rivers. A total of 12 replicate plots (50 cm×50 cm) were clipped to ground level at each site, dried at 80°C and weighed. Samples were analyzed for total C and N using a dry combustion analyzer (LECO, St Joe Michigan, USA). Data are reported as total biomass Mg ha^−1^ and total biomass N in kg ha^−1^.

### Statistical Analyses

Data were analysed using analysis of variance, Pearson correlations, and linear and non-linear regression. All data analyzed using SPSS ver 19.

## Results

**Figure 1:**
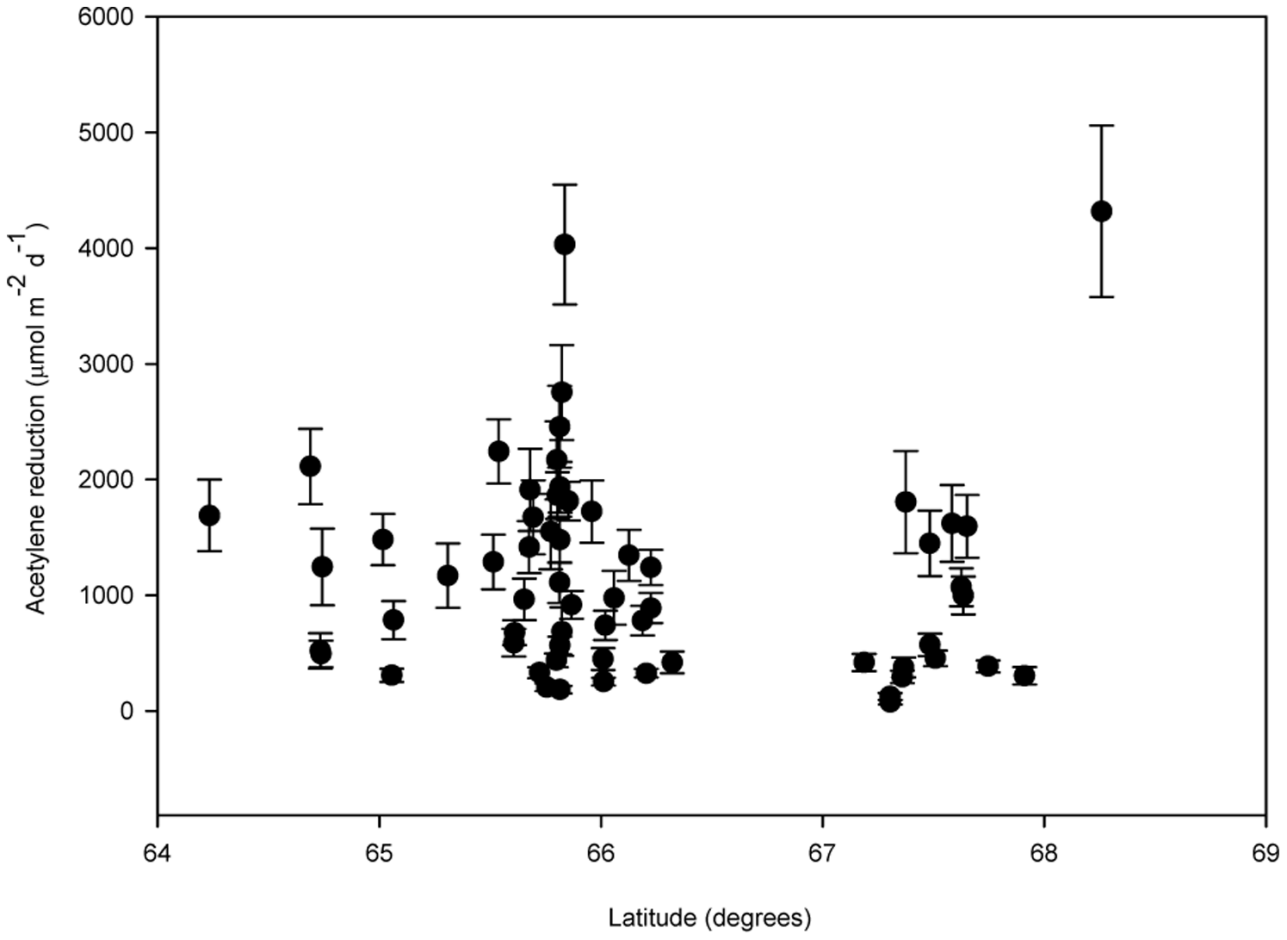
Survey of nitrogenase activity in subarctic alluvial meadows at 71 total wet meadow sites along 10 individual river systems in northern Sweden (see SI). The horizontal solid line at 1150 µmol m^−2^ d^−1^ represents the average for all 71 sites. Bars represent one standard error of the mean acetylene reduction value at each site (n = 12).

Nitrogenase activity was in general high along all 10 rivers and most of the 71 alluvial sites investigated across northern Sweden (Fig. 1; Fig. S1). Average acetylene reduction values ranged from 70 to 4,330 µmol m^−2^ d^−1^ and an overall average nitrogenase activity for the 71 sites of 1,149 µmol m^−2^ d^−1^ was discovered. At these high latitudes (64–68°N), there was no specific relationship between latitude and N_2_ fixation rates when evaluated across all sites (Fig. 1). We recorded relatively low rates of N_2_ fixation in alluvial meadows of large river systems in the south of Sweden and Norway and found that N_2_ fixation rates at southern latitudes (62–65° N) decreased with decreasing latitude.

**Figure 2:**
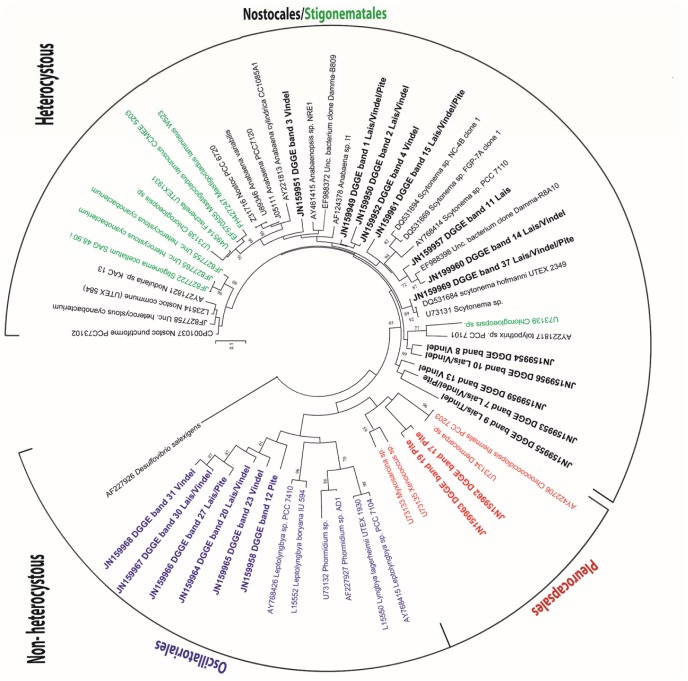
Phylogenetic affiliation of sequences retrieved using cyanobacterial specific *nif*H genes as targets combined with DGGE analyses of sediment associated DNA samples from wetland meadows along three major rivers (Vindel, Pite, and Lais) in northern Sweden. The phylogram was constructed using the maximum likelihood distance method with TrNIG model. Sequences obtained here are given in bold. Each DGGE band sequence is designated by their accession number, a code and the name of the river/s were it was collected. The numbers given at the nodes represent bootstrap values.

**Figure 3:**
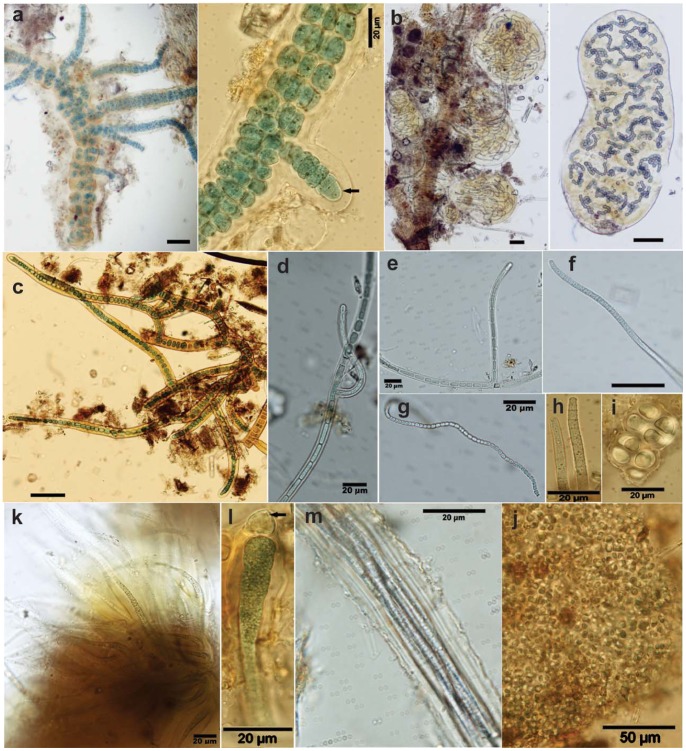
Representative cyanobacterial morphotypes identified in wetland meadows along the Lais, Vindel and Pite Rivers in northern Sweden. **a) **A filamentous, multiseriate cyanobacterium of the *Stigonema* type with multiple, true T-branches within a sheath (Vindel River). The right-hand micrograph shows a detail of a branching filament, with granulated vegetative cells, and a nitrogen-fixing heterocyst at the end of one branch (arrow). **b)** Rounded *Nostoc*-like colonies associated with vegetation debris. The right-hand part shows the winding heterocystous filaments embedded within a gelatinous matrix/sheath (Vindel River). **c)–e)** False branching uniseriate filamentous cyanobacteria of the *Scytonema*-type; c) within a distinct yellow-brown sheath (Lais and Vindel Rivers). **f)–h)** Filamentous non-heterocystous cyanobacteria of the *Oscillatoria*-type (Vindel and Pite Rivers) and in g) thin filaments of the *Leptolyngbya*-type.** i)–j)** Unicellular cyanobacterial colonies (Vindel and Pite Rivers).** k)–l)** Heterocystous cyanobacteria of the *Calothrix* type with tapering filaments within yellow-brown sheaths; k) numerous clustered filaments, and l) part of a filament with a heterocyst at the widening basal part (arrow) (Vindel River). **m) **Densely packed filaments of the non-heterocystous *Microcoleus*-type organized within a common sheath (Vindel River). Scale bar represents 50 µm, if not otherwise given.

Initial observations led us to focus on evaluation of cyanobacteria as the primary drivers of N fixation in these alluvial systems. Willow and sedge roots and lower stems as well as moss shoots were frequently covered with a cyanobacterial biofilms. Surface detritus was also clearly identifiable as being colonized by cyanobacteria. The tentative identification of diazotrophic cyanobacterial representatives (both heterocystous and non-heterocystous genera) was analyzed based on *nif*H gene phylogenies (Fig. 2) and verified by light and fluorescence microscopy (Fig. 3). The majority of the *nif*H sequences obtained were at most of the river sites examined and were affiliated with a mixed cluster of Nostocales and Stigonematales heterocystous phylotypes (belonging to cyanobacteria from section IV and V, respectively) further verified by LM (Fig. 3a–e; 3k–l). Genera related to *Nostoc/Anabaena* (Fig. 2, Fig. 3b), the false branching *Scytonema* and *Tolypothrix* (Fig. 2, Fig. 3c–e) and the true branching genus *Stigonema* were identified, as also verified by 16S rRNA gene analyses (Fig. S3). The latter is possibly related to the Stigonematales/*Stigonema ocellatum* recently found in a geographically related moss ecosystem [39]. Filamentous non-heterocystous cyanobacterial *nif*H sequences, clustering with members of the Oscillatoriales (section III) (Fig. 2), were also present at all three river sites examined as also revealed by 16S rRNA gene sequence (Fig. S4) and light microscopy (Fig. 3f–h) analyses. These genera were intermixed with aggregated unicellular cyanobacterial phenotypes from Pleurocapsales section II (Fig. 3i–j).

**Figure 4:**
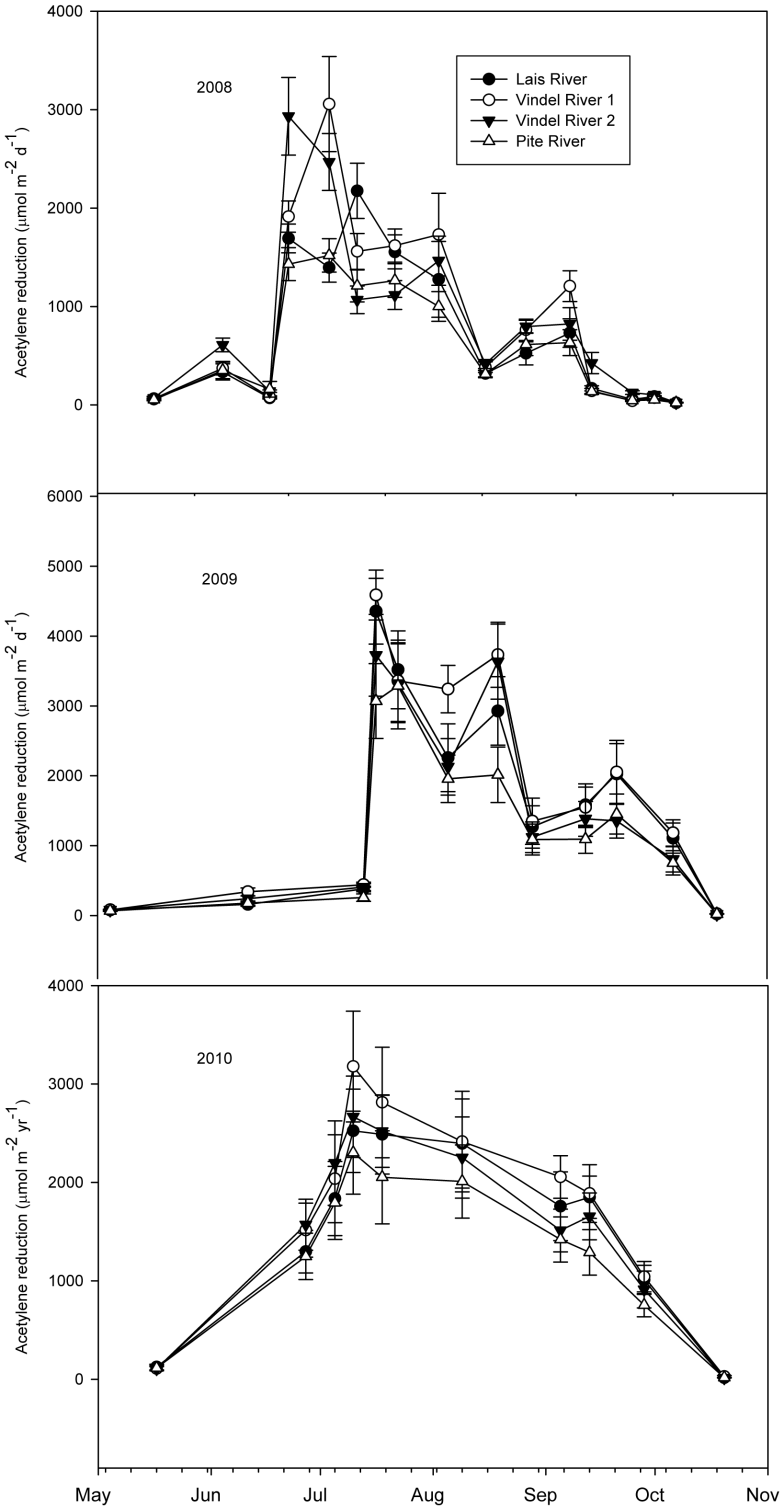
Nitrogenase activity in four intensively sampled alluvial meadow sites along three major rivers (Vindel, Pite, and Lais) in Northern Sweden assayed from May 2008 to November 2010. Each data point represents average nitrogenase activity (acetylene reduction) across the four sampling areas and 12 subsamples at each site. Bars represent one standard error for the average at each site (n = 12).

To accurately evaluate annual N_2_ fixation patterns and yields, we conducted an intensive *in-situ* field assessment of N_2_ fixation rates at four alluvial meadow sites located at three rivers in northern Sweden (Fig. 4). Weighted estimates of N_2_ fixation rates for the four sites were found to average 16.6 (+/−2.5) kg N ha^−1^ yr^−1^ in 2008, 28.8 (+/−2.4) kg N ha^−1^ yr^−1^ in 2009 and 28.0 (+/−2.5) kg N ha^−1^ yr^−1^ in 2010, with an average N_2_ fixation rate for the three year period being approximately 24.5 kg N ha^−1^ yr^−1^.

**Figure 5:**
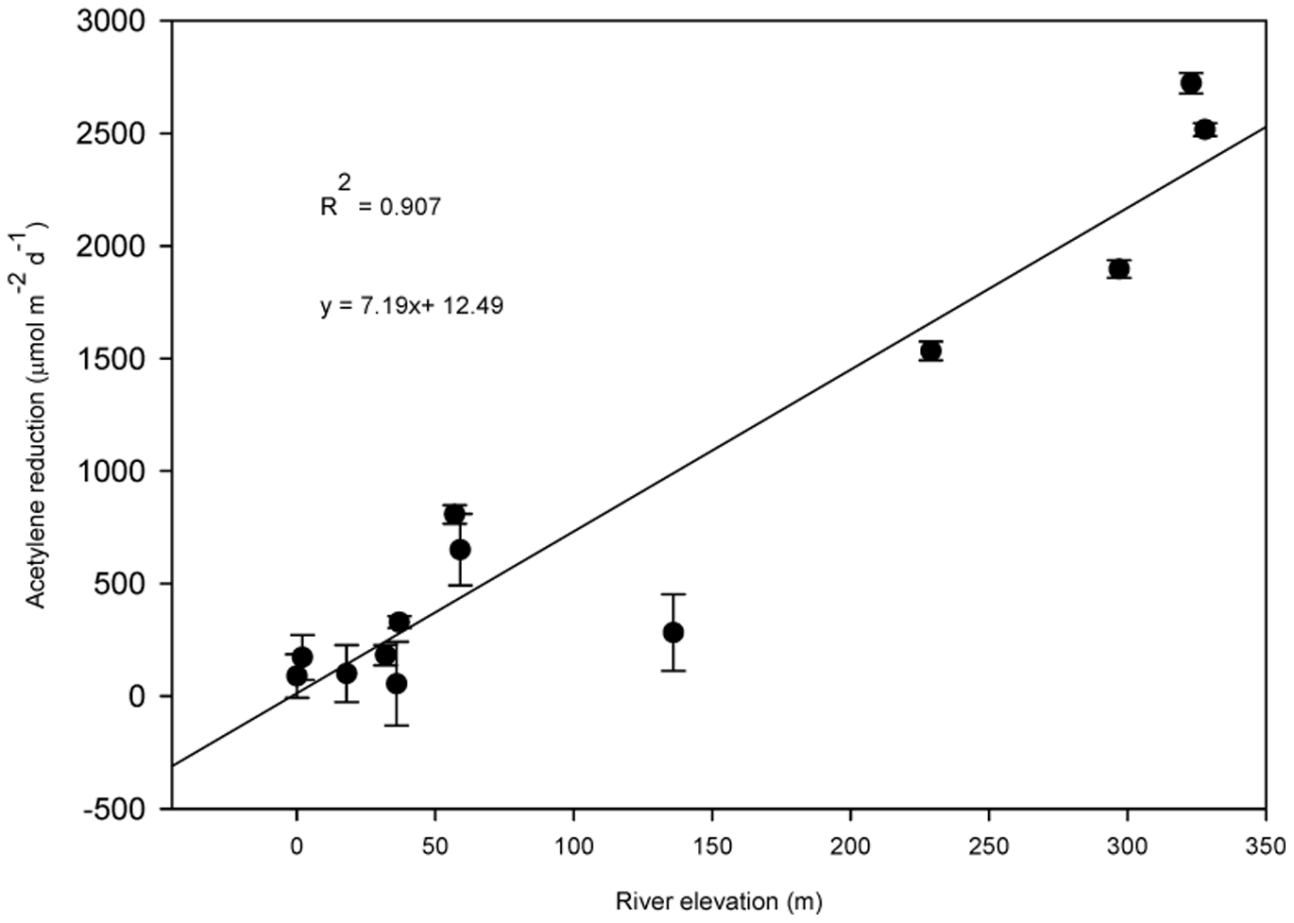
Nitrogenase activity in thirteen individual alluvial meadow sites at different river elevations along the Torne River in Northern Sweden. Lower river elevations are associated with more dense populations, increasing agricultural development and increasing wastewater of effluent from communities. Each data point represents the mean of seven sampling dates with samples collected during the summer of 2009 with 12 subsamples collected at each site on each sample date, bars represent one standard error (n = 7).

**Figure 6:**
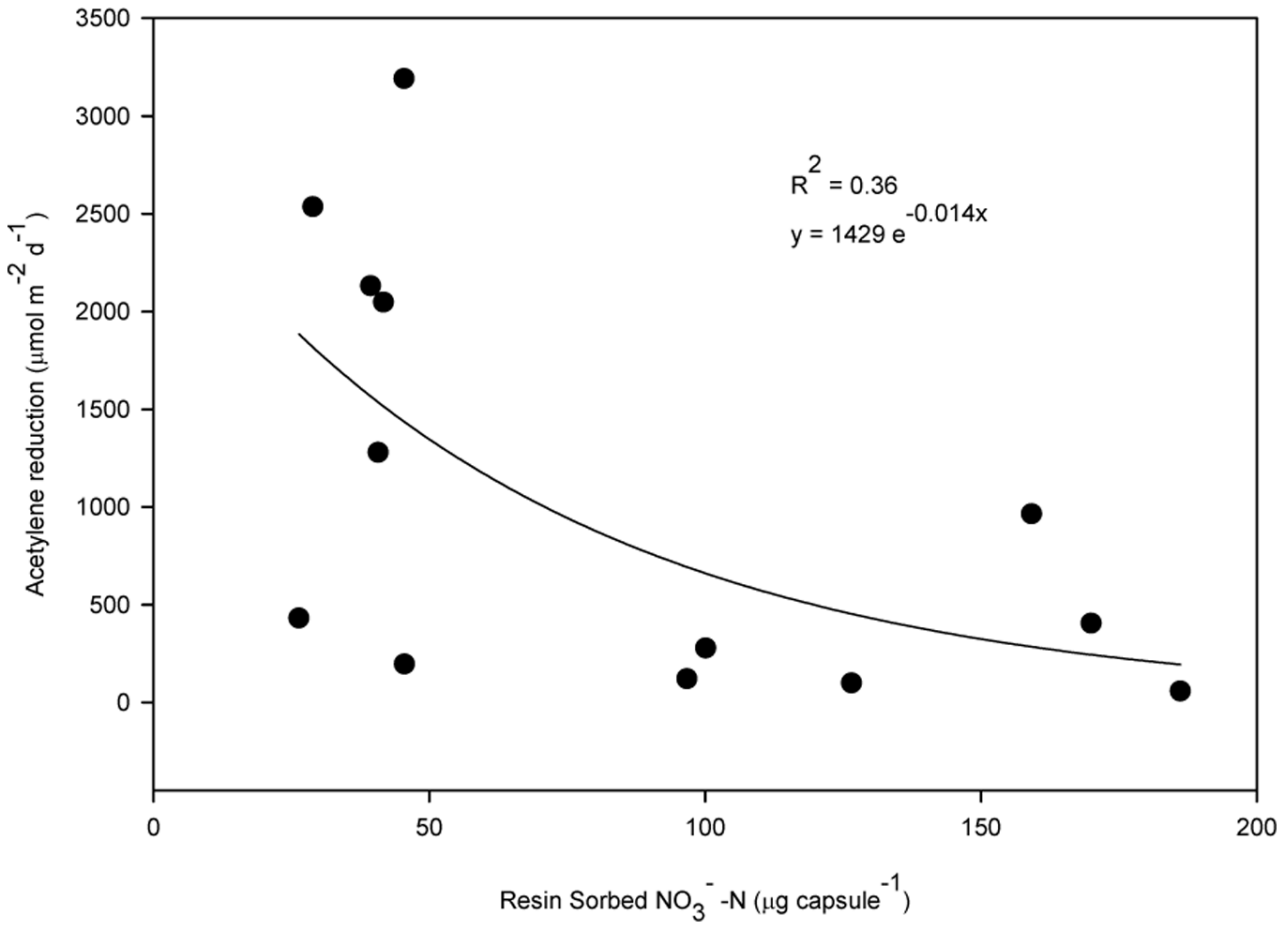
Nitrogenase activity and resin sorbed inorganic N (NH_4_^+^-N and NO_3_^−^ −N) at fourteen different alluvial meadow sites at different river elevations along the Torne River in northern Sweden. Inorganic N was collected on ionic resin capsuel tethered and buried in alluvial sediments for nine months (September 2009 to June 2010). Resins buried in areas of greater human population density tended to have higher inorganic N concentrations (inorganic N was also related to river elevation following an exponential decline with increasing elevation).

Intensive sampling along one of the rivers (Torne River) revealed a distinct decline in N_2_ fixation with decreasing river elevation (Fig. 5), roughly following a pattern of increasing development and anthropogenic N pollution. To evaluate the notion that N_2_ fixation rates in alluvial meadows is being influenced by anthropogenic N sources, we installed ionic resin capsules in surface sediments at all sampling sites in the summer period. Inorganic N (NH_4_^+^ and NO_3_^−^) were allowed to accumulate on the resins over a three month period (June–September 2010). Resin inorganic N was found to be negatively correlated with N_2_ fixation (Fig. 6).

Fodder harvest was found to remove approximately 80–140 kg N ha^−1^ yr^−1^ suggesting that biological N_2_ fixation would supply approximately 10–35% of total harvest N removal. However, in a separate controlled experiment along one river segment, N fertilization treatments nearly eliminated nitrogenase activities whereas clipping of the alluvial meadow vegetation promoted nitrogenase activity (Fig. S5).

## Discussion

The results presented here provide the first extensive effort to quantify N_2_ fixation across alluvial floodplains of boreal and subarctic ecosystems. The high rates of N_2_ fixation in these ecosystems are impressive for any ecosystem, but are particularly striking considering the climatic conditions at these high latitudes. The scale of these river systems and size of the river floodplains require reconsideration of the low levels of biological N_2_ fixation described in boreal ecosystems [17], [40] and beg consideration in landscape scale geochemical assessments [21].

These rates of N_2_ fixation are similar to rates reported for Cyprus swamps and somewhat less than rates reported for Everglades wetlands which have a 365 d growing season and water temperatures above 20°C [41]. The similarity of N_2_ fixation rates between our findings and those of subtropical systems is stunning, given the relatively short growing season (∼200 d) and low average annual temperatures (∼1°C) in alluvial meadows of boreal and subarctic ecosystems of northern Sweden. The broad geographic range of sample sites, combined with the detailed seasonal sampling and sheer numbers of individual analyses gives us a great degree of confidence in the values reported herein. The extremely high rates of acetylene reduction (calibrated with ^15^N_2_) combined with the consistency of these high rates of activity in all pristine locations over three years further supports the notion that these are not anomalous values.

Nitrogen pollution appears to exert a strong down regulating effect on N_2_ fixation in the alluvial meadows. This was clearly evident along the Torne River where increasing development and agricultural production with decreasing river elevation was linked with decreasing N_2_ fixation (Fig. 5). Agricultural development is known to increase N loading of rivers which in turn down-regulates N_2_ fixation in river systems [30]. This was further evidenced by significant effect of decreasing latitudes on N_2_ fixation in the south of Sweden where N deposition rates and river N enrichment from human effluent and agricultural practices are greater to the south [28].

We have not yet evaluated what portion of the fixed N remains in the meadows *versus* how much is lost via denitrification or down the river to the litoral N spiral, but given that the surface organic horizons (to 30 cm) in 12 meadow samples averaged 3,394 kg N ha^−1^ (+/−350), it is likely that a notable portion of the fixed N is incorporated into the associated plants and retained on site annually. Other possible contributions to new N deposits, besides N_2_ fixation, include atmospheric deposition, spawning fish, bird droppings and agricultural inputs. However, the δ^15^N signature of background detrital/biofilm samples averaged −0.37 (+/−0.06 standard error, n = 7) which lacks a strong marine isotopic signature [39] and falls below the majority of values reported for N atmospheric deposition (associated with fossil fuel burning) in the Arctic [42] and those associated with agricultural pollution [43].

Phylogenetic (Fig. 2) and light microscopy (Fig. 3) analyses demonstrate the common occurrence and rich diversity of cyanobacteria in alluvial floodplains in northern Fennoscandia. The cyanobacteria were primarily free-living, existing as ‘biofilms’ on live mosses, on willow roots, and on plant detritus. Hence, a variety of free-living cyanobacterial pheno- and genotypes with potential diazotrophic capacities were present in alluvial surface samples from the three rivers examined. Although some contribution by diazotrophic heterotrophic bacteria cannot be excluded, the many fold larger cell sizes and common occurrence of cyanobacteria stress their importance as N_2_ fixers in these systems.

Harvesting of hay in replicated plots at one location demonstrated an increase in N_2_ fixation with fodder harvest (Fig. S5). This may be a consequence of an increased light regime which would support growth and diazotrophy of the photoautotrophic cyanobacteria, or it may be a result of decreased detrital N input to surface sediments. This suggests that forage harvest losses may, at least partially, be counteracted by increases in N_2_ fixation and further enhanced by multiple harvests in a given season.

Headwater streams are thought to be important sinks of N via nutrient spiralling [44], inorganic N uptake and denitrification [30]. Our results, however, demonstrate that floodplains associated with upper reaches of large subarctic river systems could provide a significant annual input of biologically fixed N (via diazotrophs such as cyanobacteria) to river ecosystems. These findings shed light on biological N_2_ fixation as a potentially important natural ecological driver of river productivity in pristine Arctic and subarctic alluvial meadows, and fill an large gap in our knowledge of biological N_2_ fixation in alluvial floodplains [21], and help explain early settlement and the evolution of farming in this remote part of the world. Our findings also have relevance for the sustained growth in other ephemerally submerged ecosystems in less polluted globally wide-spread tropical and temperate ecosystems and may help in the design of future sustainable agricultural systems based on improved knowledge of historical and natural processes. Finally, current river N models have been identified to suffer from inaccuracies which may partially relate to the fact that these models almost exclusively ignore biological N_2_ fixation as a significant N input [44]. These data may help inform modelling efforts as an N input to riverine ecosystems.

## Supporting Information

Figure S1 **Names and locations of 10 rivers used in the survey of N_2_ fixation at various wet meadows along rivers in northern Sweden.** Segments of the rivers Vindel, Lais, Malo, Arjeplog, Pite, Kalix, Lanio, Torne, Tärendo, Konkänä were sampled and analyzed for nitrogenase activity with a subset analysed for cyanobacterial community composition.
(TIF)

Figure S2 **Areal image of an alluvial meadow examined at Vindel River. An example of a natural** ***Salix*** **spp.,** ***Carex*** **spp. meadow with hay drying huts in managed meadow in the background (photo T.H. DeLuca).**
(TIF)

Figure S3 **Phylogenetic affiliation of sequences retrieved using cyanobacterial specific 16S rRNA genes as targets combined with DGGE analyses of sediment associated DNA samples from wetland meadows along three major rivers in northern Sweden: Lais, Vindel and Pite Rivers.** The phylogram was constructed using the maximum likelihood distance method with GTRIG model. Sequences obtained here are given in bold. Each DGGE band sequence is designated by its accession number, a code and the name of the river/s were it was present. The numbers associated with the nodes represent bootstrap values.
(TIF)

Figure S4 **Nitrogenase activity in alluvial meadows as influenced by latitude for rivers in Southern to central Sweden from 62° to 65° North.**
(TIF)

Figure S5 **Nitrogenase activity as influenced by N fertilizer treatments (5 or 25 kg N ha^−1^ as NH_4_NO_3_ applied in four separate doses over a 30 d period) or clipping and removal of vegetation at an alluvial meadow site along one of the major rivers (Vindel River) in northern Sweden.**
(TIF)
