# Supplementary figures and images for: Diazotrophy in Alluvial Meadows of Subarctic River Systems
Source: PLoS One. 2013 Nov 6;8(11):e77342. doi: 10.1371/journal.pone.0077342 (PMC3819356; doi:10.1371/journal.pone.0077342)

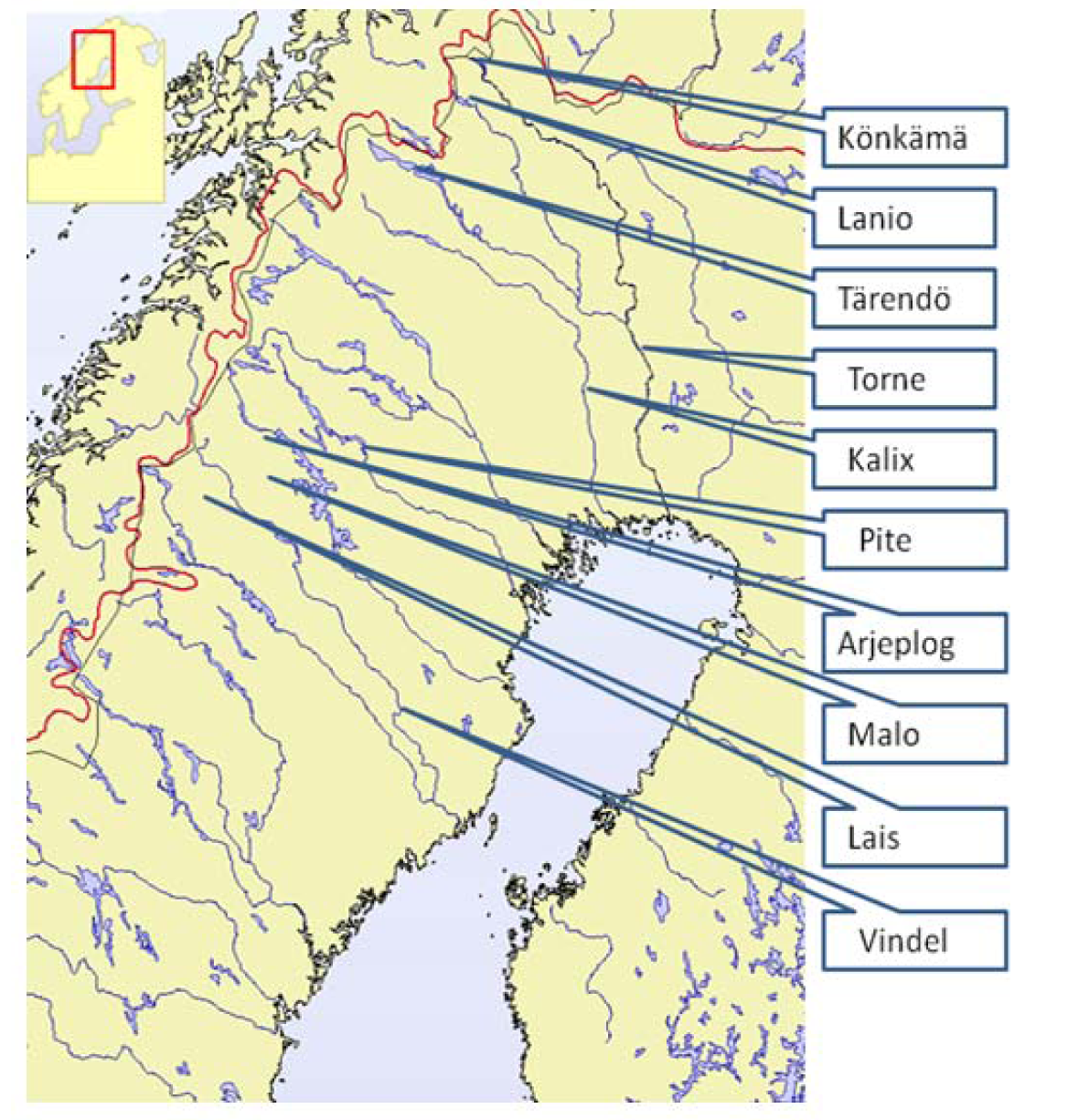

Supplement: Figure S1 — Names and locations of 10 rivers used in the survey of N2 fixation at various wet meadows along rivers in northern Sweden. Segments of the rivers Vindel, Lais, Malo, Arjeplog, Pite, Kalix, Lanio, Torne, Tärendo, Konkänä were sampled and analyzed for nitrogenase activity with a subset analysed for cyanobacterial community composition. (TIF) [file pone.0077342.s001.tif]

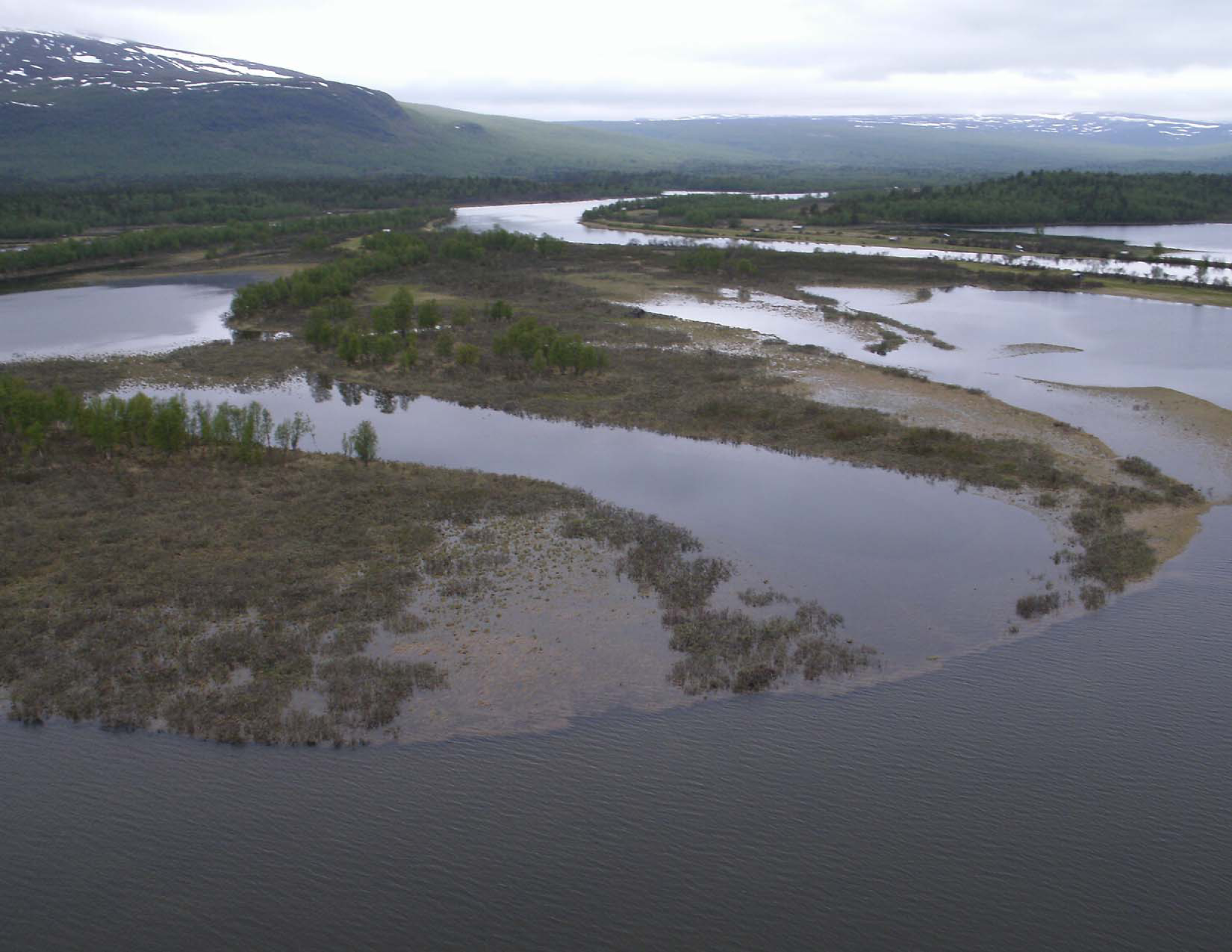

Supplement: Figure S2 — Areal image of an alluvial meadow examined at Vindel River. An example of a natural Salix spp., Carex spp. meadow with hay drying huts in managed meadow in the background (photo T.H. DeLuca). (TIF) [file pone.0077342.s002.tif]

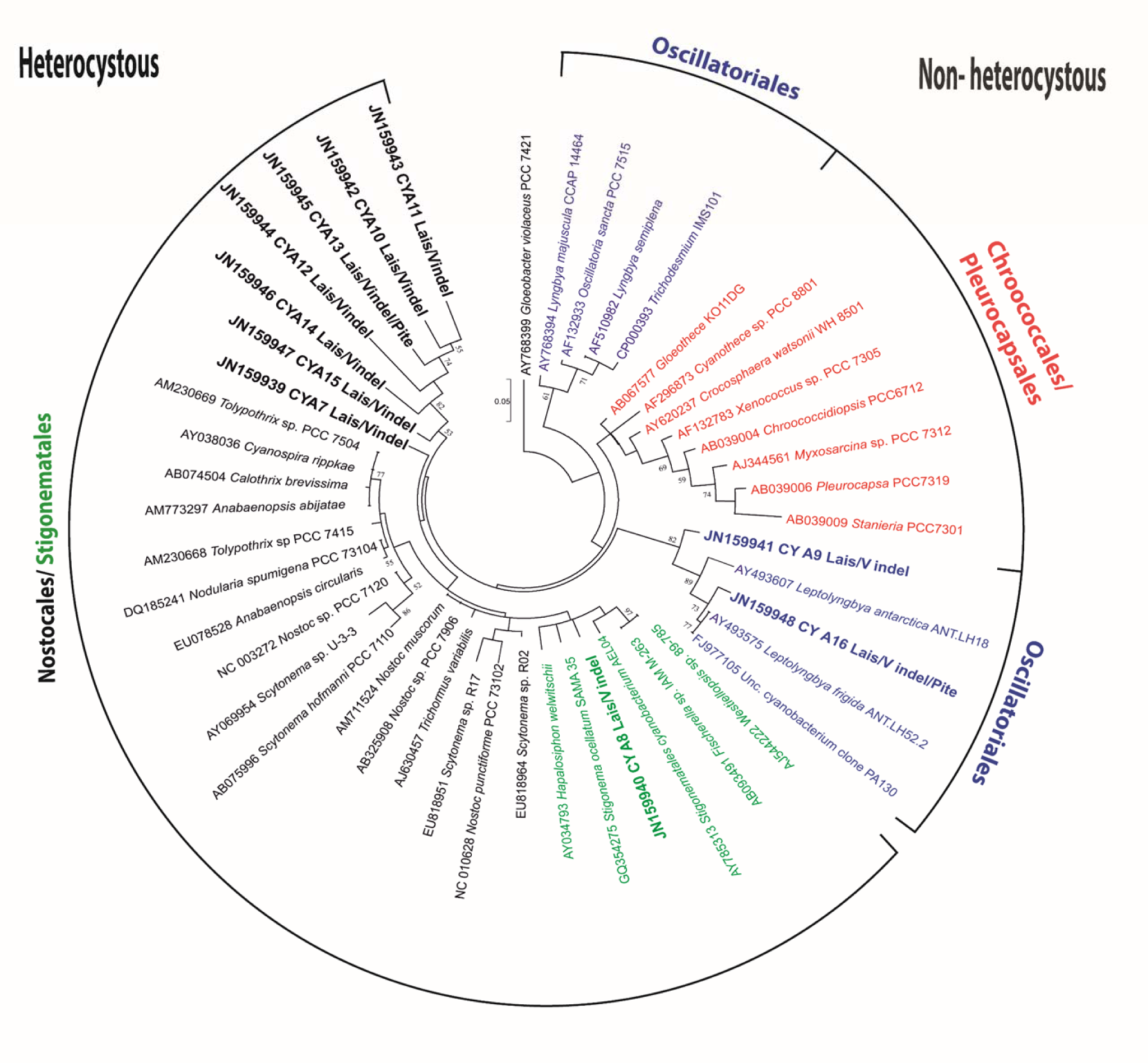

Supplement: Figure S3 — Phylogenetic affiliation of sequences retrieved using cyanobacterial specific 16S rRNA genes as targets combined with DGGE analyses of sediment associated DNA samples from wetland meadows along three major rivers in northern Sweden: Lais, Vindel and Pite Rivers. The phylogram was constructed using the maximum likelihood distance method with GTRIG model. Sequences obtained here are given in bold. Each DGGE band sequence is designated by its accession number, a code and the name of the river/s were it was present. The numbers associated with the nodes represent bootstrap values. (TIF) [file pone.0077342.s003.tif]

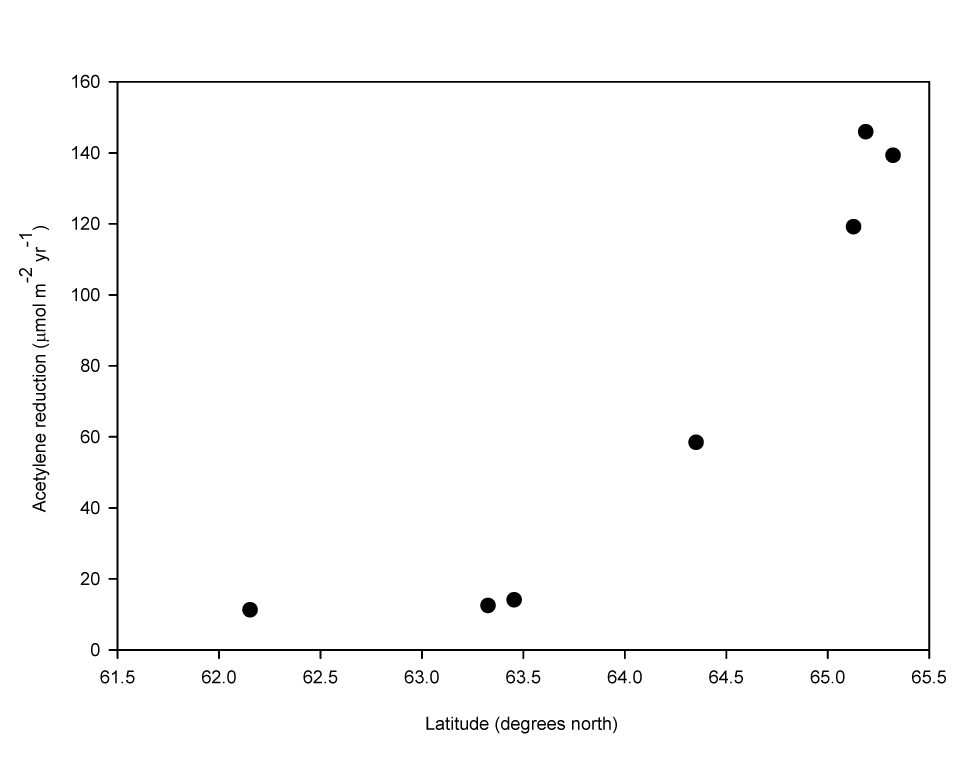

Supplement: Figure S4 — Nitrogenase activity in alluvial meadows as influenced by latitude for rivers in Southern to central Sweden from 62° to 65° North. (TIF) [file pone.0077342.s004.tif]

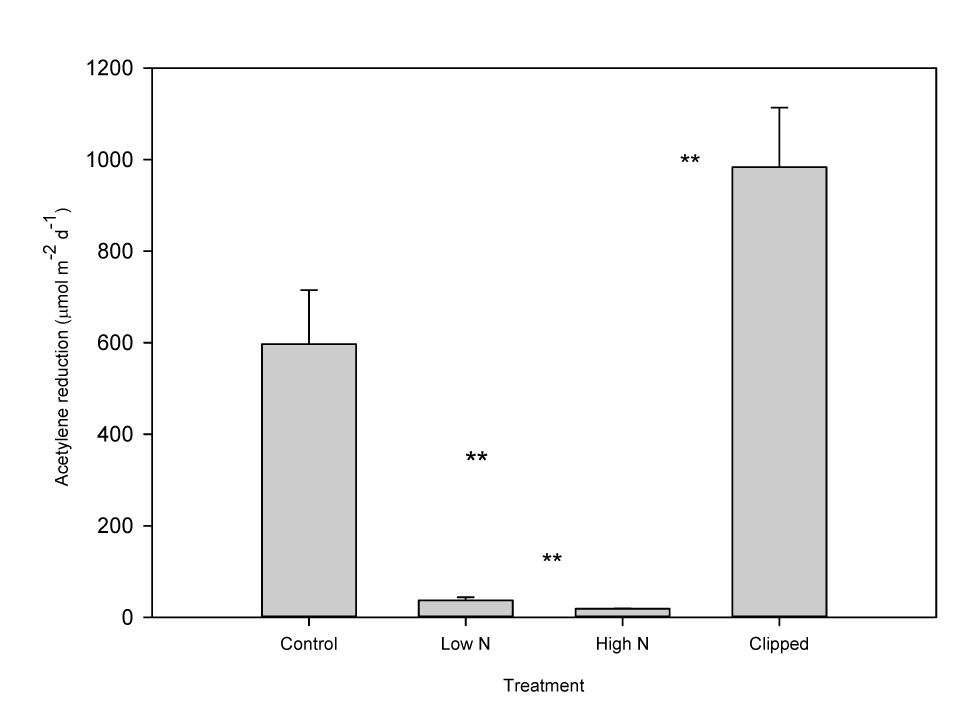

Supplement: Figure S5 — Nitrogenase activity as influenced by N fertilizer treatments (5 or 25 kg N ha−1 as NH4NO3 applied in four separate doses over a 30 d period) or clipping and removal of vegetation at an alluvial meadow site along one of the major rivers (Vindel River) in northern Sweden. (TIF) [file pone.0077342.s005.tif]
